# Toxicokinetics of 11 *Gelsemium* Alkaloids in Rats by UPLC-MS/MS

**DOI:** 10.1155/2020/8247270

**Published:** 2020-07-15

**Authors:** Xiuwei Shen, Jianshe Ma, Xianqin Wang, Congcong Wen, Meiling Zhang

**Affiliations:** ^1^Ruian People's Hospital, The Third Affiliated Hospital of Wenzhou Medical University, Wenzhou 325000, China; ^2^School of Basic Medicine, Wenzhou Medical University, Wenzhou 325035, China; ^3^Analytical and Testing Center, School of Pharmaceutical Sciences, Wenzhou Medical University, Wenzhou 325035, China; ^4^Laboratory Animal Center of Wenzhou Medical University, Wenzhou 325035, China

## Abstract

*Gelsemium elegans* (*Gardn. & Champ.*) *Benth.* is a plant belonging to the *genus Gelsemium* (*family Gelsemiaceae*), and its main components are alkaloids. It is a Chinese traditional medicinal plant and notoriously known as a highly toxic medicine. However, a method has not yet been found for the simultaneous detection of 11 *Gelsemium* alkaloids in rat plasma, and the toxicokinetics of 11 *Gelsemium* alkaloids after intravenous administration has not been reported. In this work, we have developed a sensitive and rapid method of ultraperformance liquid chromatography/tandem mass spectrometry (UPLC-MS/MS) for the detection of 11 *Gelsemium* alkaloids in rat plasma. The toxicokinetic behavior was also investigated, so as to provide a reference of the scientific properties of *Gelsemium elegans* and improve the efficacy and safety of drugs. Sixty-six Sprague-Dawley rats were randomly divided into 11 groups, six rats in each group. Each group was intravenously given one alkaloid (0.1 mg/kg), respectively. A Waters UPLC BEH C18 column (50 mm × 2.1 mm, 1.7 *μ*m) was used for chromatographic separation. Methanol and water (containing 0.1% formic acid) were used for the mobile phase with gradient elution. Multiple reactions were monitored, and positive electrospray ionization was used for quantitative analysis. The precision was less than 16%, and the accuracy was between 86.9% and 113.2%. The extraction efficiency was better than 75.8%, and the matrix effects ranged from 88.5% to 107.8%. The calibration curves were in the range of 0.1–200 ng/mL, with a correlation coefficient (*R*^2^) greater than 0.995. The UPLC-MS/MS method was successfully applied to the toxicokinetics of 11 *Gelsemium* alkaloids in rats after intravenous administration (0.1 mg/kg for each alkaloid). The results of the toxicokinetics provide a basis for the pharmacology and toxicology of *Gelsemium* alkaloids and scientific evidence for the clinical use of *Gelsemium* alkaloids.

## 1. Introduction

About 12,000 kinds of alkaloids have been found, most of which have relatively strong physiological activity. Many alkaloids have been widely used in the field of medicine [1]. For example, morphine plays an analgesic role in opium [2]. Codeine, also an alkaloid, can relieve a cough, and ephedrine plays an antiasthmatic role in ephedra [3]. Chinese herbal medicines often contain poisonous alkaloids, which have the functions of antitumor, antivirus, antiplatelet aggregation, anti-inflammatory, antiarrhythmia, and antihypertension. These officinal values have attracted great attention from the international medical and pharmaceutical circles [4]. *Gelsemium elegans* (*Gardn. & Champ.*) *Benth.* is a plant belonging to the *genus Gelsemium* (*family Gelsemiaceae*), whose main components are alkaloids. It is warm, pungent, bitter, and poisonous. It is mainly distributed in the Fujian and Zhejiang provinces in China. It is a Chinese traditional medicinal plant and also a world-famous highly toxic medicine [5]. In China, it is mainly used for external application because of its high toxicity. It can dispel wind and blood stasis, reduce swelling, relieve pain, and kill insects. In recent years, pharmacological studies have shown that *Gelsemium elegans* has many functions, including analgesic effects, sedation, anti-inflammation, mydriatic effects, antitumor, reduced heart rate, heart contraction inhibition, increasing blood pressure, and improving immunity [6, 7]. The antitumor effect of *Gelsemium elegans* provides an attractive prospect for research and development. Indole alkaloids are the main chemical components in *Gelsemium elegans* and the main source of its toxicity. However, as the toxic dose is close to the effective dose, poisoning events of the *Gelsemium elegans* are common in clinical practice. Seventeen kinds of poisonous alkaloids have been isolated from *Gelsemium elegans*, among which koumine is the most abundant, followed by gelsemine. As the alkaloid types and contents from different places and different plant parts of *Gelsemium elegans* are slightly different, there are currently not many reports regarding the toxicokinetics of *Gelsemium* alkaloids *in vivo* [8–10]. It has been a popular research topic to analyze alkaloids from different sources in complex systems quickly, sensitively, and reliably. UPLC-MS/MS, because of its flexibility, exhibits advantages of good sensitivity, excellent separation ability, wide application range, and strong specificity, which is why it is widely used in alkaloid analysis [11–14].

There have been methods reported for the determination of *Gelsemium* alkaloids, including high performance liquid chromatography-tandem (HPLC) [15, 16], ultraperformance liquid chromatography-quadrupole-time of flight mass spectrometry (UPLC-Q-TOF/MS) [17, 18], liquid chromatography/tandem mass spectrometry (LC/MS/MS) [19–23], and ultraperformance liquid chromatography/tandem mass spectrometry (UPLC-MS/MS) [24–28] *in vivo*. However, a method has not been found for the simultaneous determination of 11 *Gelsemium* alkaloids in rat plasma, and the toxicokinetics of 11 *Gelsemium* alkaloids (humantenirine, humantenine, akuammidine, gelsevirine, rankinidine, n-methoxyanhydrovobasinediol, gelsenicine, gelsemine, koumine, koumidine, and sempervirine) after intravenous administration has not been reported. In this work, a sensitive and rapid method of UPLC-MS/MS was developed for the determination of 11 *Gelsemium* alkaloids in rat plasma, and the toxicokinetic behavior was investigated, so as to provide a scientific understanding of *Gelsemium elegans* and improve the efficacy and safety of drugs.

## 2. Materials and Methods

### 2.1. Materials and Reagents

**Figure 1:**
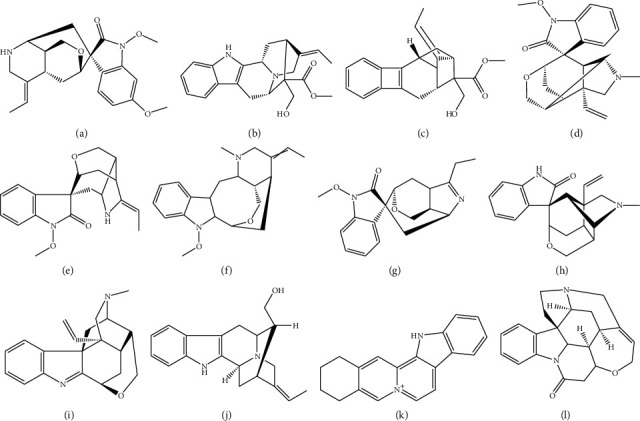
Chemical structures of humantenirine (a), humantenine (b), akuammidine (c), gelsevirine (d), rankinidine (e), n-methoxyanhydrovobasinediol (f), gelsenicine (g), gelsemine (h), koumine (i), koumidine (j), sempervirine (k), and strychnine (IS, l).

Humantenirine, humantenine, akuammidine, gelsevirine, rankinidine, n-methoxyanhydrovobasinediol, gelsenicine, gelsemine, koumine, koumidine, sempervirine (all >98%, Figure 1), and the internal standard strychnine (IS, all >98%) were purchased from Chengdu Mansite Biotechnology Co., Ltd (Chengdu, China). HPLC grade methanol and acetonitrile were obtained from Merck Company (Darmstadt, Germany). Ultrapure water (resistance > 18 m*Ω*) was prepared by Millipore Milli-Q (Bedford, USA).

### 2.2. Instrumentation and Conditions

An ACQUITY H-Class UPLC and XEVO TQ-S micro triple quadrupole mass spectrometer was obtained from Waters Corp. (Milford, MA, USA).

Eleven *Gelsemium* alkaloids (humantenirine, humantenine, akuammidine, gelsevirine, rankinidine, n-methoxyanhydrovobasinediol, gelsenicine, gelsemine, koumine, koumidine, and sempervirine) and IS were separated using a Waters UPLC® BEH C18 column (50 mm × 2.1 mm, 1.7 *μ*m) maintained at 40°C. The mobile phase consisted of methanol and water (containing 0.1% formic acid). The gradient elution was as follows: from 0 to 0.2 min, the methanol was kept at 10%; from 0.2 to 2.0 min, methanol increased from 10% to 80%; from 2.0 to 2.5 min, the methanol was kept at 80%; from 2.5 to 2.8 min, methanol was changed from 80% to 10%; and at last, methanol was kept at 10% for 2.2 min. The flow rate was set at 0.4 mL/min.

**Figure 2:**
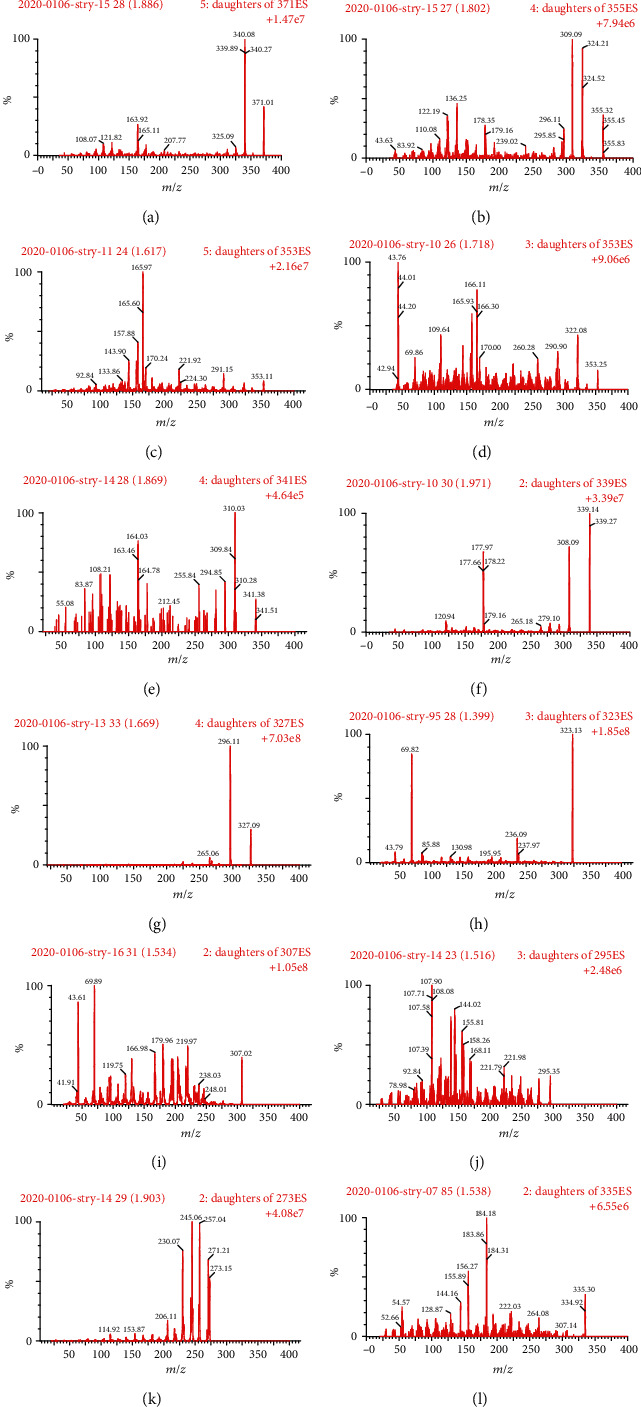
Mass spectra of humantenirine (a), humantenine (b), akuammidine (c), gelsevirine (d), rankinidine (e), n-methoxyanhydrovobasinediol (f), gelsenicine (g), gelsemine (h), koumine (i), koumidine (j), sempervirine (k), and strychnine (IS, l).

Nitrogen was used for the cone gas (50 L/h) and desolvation gas (900 L/h). The capillary voltage was 2.5 kV, the source temperature was 150°C, and the desolvation temperature was 450°C. The employed collision gas for fragmentation in the multiple reaction monitoring (MRM) mode was argon. The MRM mode (Table 1) was used for quantitative analysis in the electrospray ionization (ESI) positive interface (Figure 2).

### 2.3. Stock Solutions

Stock solutions of 100 *μ*g/mL each of humantenirine, humantenine, akuammidine, gelsevirine, rankinidine, n-methoxyanhydrovobasinediol, gelsenicine, gelsemine, koumine, koumidine, sempervirine, and IS were prepared in methanol. The working standard solutions of each of the 11 *Gelsemium* alkaloids were prepared by dilution of the stock solution with methanol. The stock solutions and working standard solutions were stored at 4°C.

The calibration standards were prepared by spiking blank rat plasma with appropriate amounts of the 11 *Gelsemium* alkaloid working standard solutions. Calibration plots of each of the 11 *Gelsemium* alkaloids were constructed in the 0.1-200 ng/mL range for plasma (0.1, 0.5, 2, 10, 20, 50, 100, and 200 ng/mL). Quality-control (QC) samples (0.4, 18, and 180 ng/mL) were prepared in the same manner as the calibration standards.

### 2.4. Sample Preparation

In a 1.5 mL centrifuge tube, an aliquot of 50 *μ*L plasma was added; then, 150 *μ*L acetonitrile (containing IS 20 ng/mL) was added, and the vortex was mixed for 1.0 min, then centrifuged (14900 g) for 10 min, and then, the supernatant (2 *μ*L) was injected into the UPLC-MS/MS system for analysis.

### 2.5. Method Validation

Calibration curves were established by analyzing calibration samples on three different days. The relationship between the peak area ratio of 11 alkaloids and the concentration of the analyte was fitted to the equations. In the concentration range of 0.1-200 ng/mL, linear regression was performed using the weighting factor (1/*x*) of the inverse concentration. LLOQ was calculated as the baseline of blank plasma plus the concentrations of 11 *Gelsemium* alkaloids added, and the final deviation should be within ±20%.

Precision and accuracy were assessed by measuring six replicate QC samples within three days of validation. Precision was expressed by the coefficient of variation (CV). Accuracy was determined as the extent to which the mean value corresponded to the true value.

The selectivity of this method was evaluated by analyzing six batches of blank rat plasma, blank plasma supplemented with 11 alkaloids and IS, and one rat plasma sample.

To evaluate matrix effects, blank rat plasma was extracted, and then, 0.4, 18, and 180 ng/mL of analyte were added. Then, the corresponding peak area was compared to the peak area of the pure standard solution at the same concentration, and this peak area ratio was defined as the matrix effect.

The extraction efficiency was evaluated by comparing the peak area of the extracted QC sample with the peak area (*n* = 6) of the reconstituted reference QC solution in the blank plasma extract.

The stability of 11 alkaloids in rat plasma was evaluated by analyzing three replicate plasma samples at 0.4, 18, and 180 ng/mL under different conditions [29]. The short-term stabilities of the samples after exposure were determined at room temperature for 2 h, and injection samples (after protein precipitation) were performed by UPLC at room temperature for 12 h. Three complete freeze/thaw (−20 to 25°C) cycles were evaluated on consecutive days after freezing/thawing stabilization. After storage at -20°C for 30 days, its long-term stability was evaluated.

### 2.6. Toxicokinetics

Sixty-six Sprague Dawley rats (male, 200-220 g) obtained from the Laboratory Animal Center of Wenzhou Medical University were randomly divided into 11 groups, six rats for each group. All experimental procedures and operating procedures were reviewed and approved by the Animal Care and Use Committee of Wenzhou Medical University. Each group was intravenously given humantenirine (0.1 mg/kg), humantenine (0.1 mg/kg), akuammidine (0.1 mg/kg), gelsevirine (0.1 mg/kg), rankinidine (0.1 mg/kg), n-methoxyanhydrovobasinediol (0.1 mg/kg), gelsenicine (0.1 mg/kg), gelsemine (0.1 mg/kg), koumine (0.1 mg/kg), koumidine (0.1 mg/kg), and sempervirine (0.1 mg/kg), respectively.

Blood samples (0.3 mL) were collected from the tail vein into heparinized tubes at 0.0833, 0.5, 1, 2, 3, 4, 6, 8, 12, and 24 h after intravenous administration and centrifuged for 10 min at 3000 g, and 100 *μ*L plasma was obtained. Toxicokinetics was analyzed by DAS software (Version 3.0, China Pharmaceutical University).

## 3. Results

### 3.1. Method Validation

Equations of the calibration curves (0.1-200 ng/mL) are listed in Table 2. The LLOQ for *Gelsemium* alkaloids were 0.1 ng/mL.

Intraday and interday precision was measured to be 16% or less. The accuracy ranged from 86.9% to 113.2%. Extraction efficiency was between 75.8% and 98.4% (Table 3).

**Figure 3:**
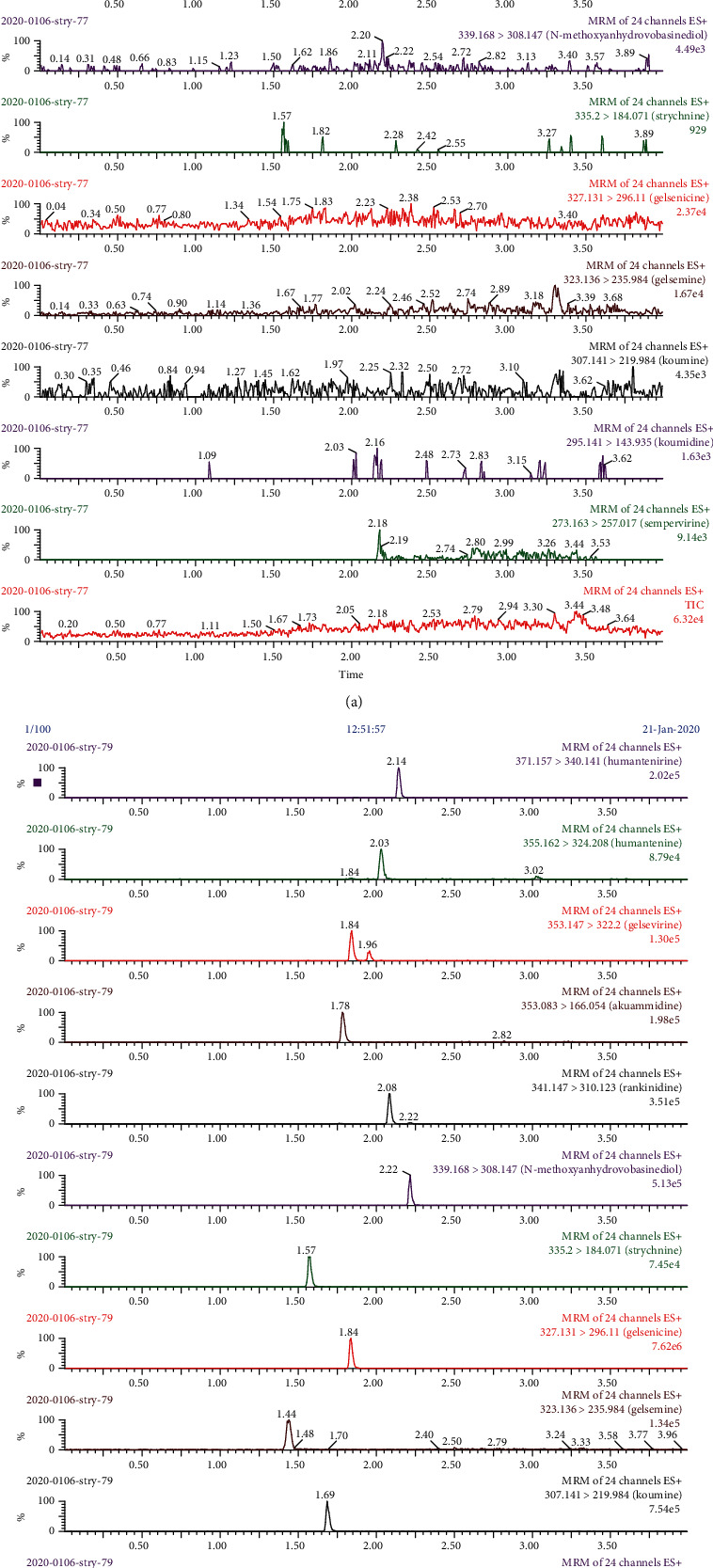
UPLC-MS/MS chromatograms of humantenirine, humantenine, akuammidine, gelsevirine, rankinidine, n-methoxyanhydrovobasinediol, gelsenicine, gelsemine, koumine, koumidine, sempervirine, and strychnine (IS) in rat plasma: (a) blank rat plasma; (b) blank rat plasma spiked with 11 Gelsemium alkaloids and IS.

Figure 3 shows the typical UPLC-MS/MS of blank rat plasma spiked with 11 *Gelsemium* alkaloids and IS. No interfering endogenous substances were observed at the retention time of the 11 *Gelsemium* alkaloids and IS.

The matrix effects were measured to be 88.5-107.8% (Table 3), indicating that matrix effects from plasma are negligible.

The stability results indicate that the 11 *Gelsemium* alkaloids are stable under the three storage conditions since the precision was within ±15% (Table 4).

### 3.2. Toxicokinetics

**Figure 4:**
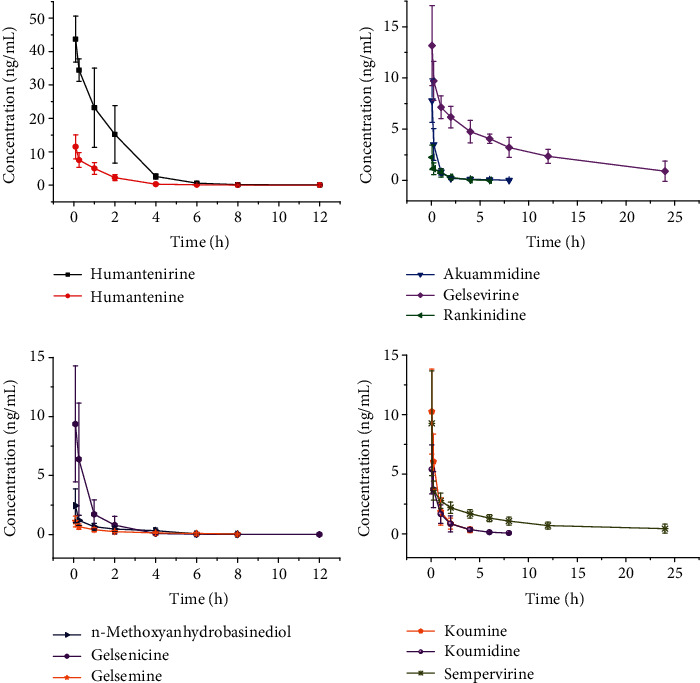
The toxicokinetic profiles of humantenirine, humantenine, akuammidine, gelsevirine, rankinidine, n-methoxyanhydrovobasinediol, gelsenicine, gelsemine, koumine, koumidine, and sempervirine in rats (*n* = 6).

The UPLC-MS/MS method was applied to the toxicokinetic study of 11 *Gelsemium* alkaloids in rats. The main toxicokinetic parameters after intravenous administration of humantenirine, humantenine, akuammidine, gelsevirine, rankinidine, n-methoxyanhydrovobasinediol, gelsenicine, gelsemine, koumine, koumidine, and sempervirine are summarized in Table 5, using noncompartment model analysis. The 11 *Gelsemium* alkaloid plasma mean concentration-time curves are shown in Figure 4.

## 4. Discussion

The feasibility of ESI in negative and positive ion modes was evaluated in this work. ESI is suitable for compounds with medium polarity to high polarity. APCI has the advantage of weak polarity to medium polarity. ESI is more suitable for the analysis of alkaloids. The sample structure contains complex nitrogen-containing heterocycles, so the positive mode should be preferred [30–32]. When the positive and negative modes were used to monitor the alkaloids simultaneously, the characteristic molecular ion peaks of 11 *Gelsemium* alkaloids had low sensitivity in the negative mode. There, ESI with the positive ion mode was used for detection of 11 *Gelsemium* alkaloids. The optimum conditions of mass spectrometric analysis of alkaloids were investigated. Through the study of spray needle voltage, drying gas temperature, dry gas pressure, capillary voltage, and impact energy, the best mass spectrometric conditions were obtained and are shown in Figure 2.

The conditions of the chromatographic separation of alkaloids were investigated. Through the study of the mobile phase, stationary phase, flow rate, and column temperature, the separation conditions were optimized. 0.1% formic acid was added into the mobile phase, and the alkaloids responded well, because the alkaloids favor ionization under the positive ion mode within acidic conditions. The mobile phase of methanol-0.1% formic acid using gradient elution almost achieved baseline separation for humantenirine, humantenine, akuammidine, gelsevirine, rankinidine, n-methoxyanhydrovobasinediol, gelsenicine, gelsemine, koumine, koumidine, and sempervirine. However, some peaks did not achieve baseline separation, and the MRM mode was used for quantitative analysis in our work, where the quantitative ion pairs were different.

Several compounds including strychnine, carbamazepine, diazepam, midazolam, and berberine were tested for IS. Strychnine was chosen as IS because of its similar mass ionization and retention time as the 11 *Gelsemium* alkaloids in a positive-ion ESI mode. Sample treatment prior to UPLC-MS/MS analysis was very important [33, 34]. An efficient and simple method of protein precipitation was used in our work. Choosing acetonitrile as the protein precipitation solvent can get better extraction efficiency and matrix effects than methanol.

The developed UPLC-MS/MS was used for the subsequent quantification of all *Gelsemium* alkaloids, which has a faster analysis time than traditional HPLC [35, 36] or LC-MS [37–39] and greatly enhances the signal intensity. It only takes five minutes to analyze a plasma sample, which can save significant time when analyzing hundreds of samples. In addition, the LLOQ of the 11 *Gelsemium* alkaloids was relatively low (0.1 ng/mL). LLOQ was calculated as the baseline of blank plasma plus the concentrations of the 11 *Gelsemium* alkaloids added, and the final deviation was within ±20%.

The 11 *Gelsemium* alkaloids are basic nitrogen-containing compounds existing in organisms. They have complex nitrogen-containing heterocycles, optical specificity, and significant physiological effects. This study has elucidated the toxicokinetic parameters of 11 *Gelsemium* alkaloids in rats. After intravenous administration, the concentrations in plasma at different time points were measured, and the toxicokinetic parameters were calculated. The half-lives of rankinidine and humaniline were the shortest, and gelsevirine was the longest, suggesting that rankinidine and humaniline absorbed and eliminated faster, had a short time to reach the peak, and had high plasma concentration. On the other hand, gelsevirine eliminated more slowly, indicating that the maintenance time of efficacy was longer. It also suggests that gelsevirine may accumulate in the body if taken for a long time [40–42]. The AUC_(0-__*t*__)_ values of humantenirine and gelsevirine were higher than the other nine alkaloids, at 73.1 ± 19.5 ng/mL∗h and 73.8 ± 12.9 ng/mL∗h, respectively. Conversely, AUC_(0-__*t*__)_ of rankinidine and gelsemine were lower than that of the other nine alkaloids, at 2.2 ± 0.8 ng/mL∗h and 1.7 ± 0.5 ng/mL∗h, respectively. The difference in the absorption of the different alkaloids was obvious.

## 5. Conclusion

The UPLC-MS/MS method has been validated for the simultaneous determination of 11 *Gelsemium* alkaloids in rat plasma, and it was successfully applied in toxicokinetic research of 11 *Gelsemium* alkaloids (humantenirine, humantenine, akuammidine, gelsevirine, rankinidine, n-methoxyanhydrovobasinediol, gelsenicine, gelsemine, koumine, koumidine, and sempervirine) in rats. The toxicokinetic results provide a basis for the study of the pharmacology and toxicology of *Gelsemium* alkaloids and provide scientific evidence for the clinical use of *Gelsemium* alkaloids.

## Figures and Tables

**Table 1:** Mass parameters for 11 *Gelsemium* alkaloids and strychnine (IS).

Compound name	Parent (*m*/*z*)	Daughter (*m*/*z*)	Cone (V)	Collision (V)
Humantenirine	371.2	340.1	38	14
Humantenine	355.2	324.2	24	16
Akuammidine	353.1	166.1	28	26
Gelsevirine	353.1	322.1	22	40
Rankinidine	341.1	310.1	14	20
n-Methoxyanhydrovobasinediol	339.2	308.1	12	14
Gelsenicine	327.1	296.1	36	15
Gelsemine	323.1	236.1	46	22
Koumine	307.1	219.9	*52*	38
Koumidine	295.1	143.9	24	25
Sempervirine	273.2	257.0	24	25
Strychnine (IS)	335.2	184.2	25	22

**Table 2:** Regression equations and correlation coefficients for 11 *Gelsemium* alkaloids in rat plasma.

Compound	Linear range (ng/mL)	Regression equation	Correlation coefficient
Humantenirine	0.1-200	*y* = 0.0125*x* − 0.0041	0.9962
Humantenine	0.1-200	*y* = 0.0092*x* − 0.0053	0.9991
Akuammidine	0.1-200	*y* = 0.0058*x* + 0.0134	0.9992
Gelsevirine	0.1-200	*y* = 0.0136*x* − 0.0060	0.9995
Rankinidine	0.1-200	*y* = 0.0117*x* + 0.0067	0.9987
n-Methoxyanhydrovobasinediol	0.1-200	*y* = 0.0153*x* + 0.0125	0.9993
Gelsenicine	0.1-200	*y* = 0.0161 *x* + 0.0010	0.9985
Gelsemine	0.1-200	*y* = 0.0018*x* − 0.0013	0.9967
Koumine	0.1-200	*y* = 0.0043*x* − 0.0014	0.9980
Koumidine	0.1-200	*y* = 0.0035*x* + 0.0025	0.9998
Sempervirine	0.1-200	*y* = 0.0048*x* + 0.0028	0.9950

*y* = peak area ratio of *Gelsemium* alkaloids versus IS; *x* = concentration of *Gelsemium* alkaloids.

**Table 3:** Accuracy, precision, extraction efficiency, and matrix effects of 11 *Gelsemium* alkaloids in rat plasma (*n* = 6).

Compound	Concentration (ng/mL)	Accuracy (%)		CV (%)		Matrix effect (%)	Recovery (%)
		Intraday	Interday	Intraday	Interday		
Humantenirine	0.1	103.2	98.4	5.7	9.9	95.4	84.0
	0.4	95.5	95.4	7.1	13.7	93.6	81.8
	18	99.4	99.2	3.0	7.5	97.3	89.9
	180	99.5	107.4	7.7	8.8	93.6	86.8
Humantenine	0.1	98.4	104.7	7.3	13.0	99.1	85.8
	0.4	97.7	98.3	2.1	5.2	98.8	89.1
	18	102.0	100.8	3.7	5.0	103.6	86.0
	180	96.5	106.3	4.7	8.4	98.1	90.0
Akuammidine	0.1	107.9	100.8	5.9	12.7	103.4	98.4
	0.4	107.3	101.7	8.0	6.7	94.1	95.4
	18	104.3	98.0	5.2	4.5	98.0	90.5
	180	96.3	99.7	5.0	4.4	107.2	98.3
Gelsevirine	0.1	106.5	109.2	5.7	12.3	98.5	89.7
	0.4	101.1	99.5	3.2	6.5	96.6	88.9
	18	99.3	95.6	5.8	9.6	99.5	93.2
	180	102.2	103.8	7.0	6.4	101.3	96.6
Rankinidine	0.1	99.3	101.5	14.9	13.3	103.2	93.8
	0.4	102.2	97.9	7.8	2.2	104.2	91.0
	18	103.2	105.0	8.8	12.6	107.7	86.5
	180	97.4	101.6	7.1	5.8	103.2	92.0
n-Methoxyanhydrovobasinediol	0.1	92.7	100.9	5.0	8.3	98.8	95.9
	0.4	106.0	97.9	4.0	4.5	97.7	89.0
	18	102.7	94.9	3.4	6.2	105.4	90.9
	180	97.4	103.8	2.5	4.0	103.4	90.6
Gelsenicine	0.1	104.5	98.2	10.9	13.8	90.6	91.6
	0.4	102.8	103.5	4.0	12.3	88.5	84.3
	18	105.1	97.0	3.3	5.3	95.1	82.2
	180	92.5	92.2	6.9	5.4	98.9	93.5
Gelsemine	0.1	86.9	96.7	16.7	12.1	97.2	82.5
	0.4	98.9	89.9	11.3	12.7	103.8	90.1
	18	106.5	91.2	13.7	8.8	107.8	86.0
	180	92.8	102.2	8.2	11.2	91.2	84.9
Koumine	0.1	113.2	91.8	6.9	15.9	94.2	95.6
	0.4	108.3	99.6	11.8	8.1	92.8	83.3
	18	99.6	91.7	8.3	11.2	91.6	82.5
	180	98.2	108.3	12.7	13.9	89.6	84.7
Koumidine	0.1	102.3	103.9	11.1	14.6	100.8	78.2
	0.4	105.5	101.2	9.5	6.1	97.7	79.8
	18	97.5	105.8	10.3	10.6	100.2	76.4
	180	99.1	96.6	5.7	8.9	94.0	82.7
Sempervirine	0.1	103.0	94.2	13.8	11.7	95.9	84.7
	0.4	96.5	104.3	6.4	8.8	97.9	77.4
	18	103.1	97.8	5.8	4.3	98.2	75.8
	180	101.0	96.7	9.7	4.3	106.0	85.6

**Table 4:** Stability of 11 *Gelsemium* alkaloids in rat plasma.

Compound	Concentration (ng/mL)	Autosampler ambient		Ambient 2 h		-20°C for 30 d		Freeze-thaw	
		Accuracy (%)	CV (%)	Accuracy (%)	CV (%)	Accuracy (%)	CV (%)	Accuracy (%)	CV (%)
Humantenirine	0.4	104.2	8.5	102.1	4.0	107.9	11.7	103.0	10.5
	18	100.7	6.3	106.1	9.1	101.5	5.9	99.3	6.1
	180	98.0	5.0	106.8	7.8	99.9	5.7	97.0	8.9
Humantenine	0.4	94.5	5.3	98.8	7.6	107.3	12.6	99.8	11.6
	18	104.4	3.6	100.8	4.6	96.5	3.5	104.0	9.6
	180	99.2	3.5	106.5	4.5	100.9	1.4	105.8	3.7
Akuammidine	0.4	102.8	7.8	98.6	8.6	111.4	11.7	90.5	14.6
	18	97.6	6.7	109.7	5.9	103.5	9.6	96.8	9.4
	180	99.1	8.1	106.7	3.1	103.6	7.4	104.3	4.6
Gelsevirine	0.4	99.5	5.4	96.9	5.8	101.3	8.5	101.1	9.2
	18	101.9	1.9	99.3	2.1	94.8	10.6	106.1	9.6
	180	96.5	4.3	103.7	4.8	93.3	10.0	99.8	7.8
Rankinidine	0.4	99.5	7.8	90.3	9.1	89.7	8.5	87.4	11.7
	18	101.8	6.0	101.4	6.3	105.5	9.8	107.4	3.4
	180	96.6	6.9	106.5	5.1	104.7	7.9	101.6	5.7
n-Methoxyanhydrovobasinediol	0.4	96.0	8.3	94.4	6.1	102.5	4.6	92.8	9.7
	18	103.8	4.3	104.1	8.5	95.3	8.9	99.8	8.3
	180	99.3	6.7	95.0	7.2	98.9	5.2	108.9	8.3
Gelsenicine	0.4	92.4	11.7	106.6	5.3	100.8	7.3	92.1	7.4
	18	98.5	6.0	93.1	11.1	94.2	10.5	105.6	11.5
	180	105.0	9.8	103.8	12.0	92.0	5.3	104.5	13.3
Gelsemine	0.4	100.8	7.4	97.2	12.6	111.4	14.0	99.7	11.5
	18	96.6	3.1	104.3	11.1	104.2	13.2	110.7	8.5
	180	95.8	2.6	104.7	10.1	103.0	8.6	91.5	3.7
Koumine	0.4	100.6	10.4	112.5	12.1	108.1	13.0	105.8	14.7
	18	97.5	8.5	108.5	7.7	88.7	11.4	104.5	14.8
	180	102.3	8.7	93.7	4.7	94.1	5.4	92.5	7.4
Koumidine	0.4	98.6	7.5	97.2	6.2	97.5	14.8	108.1	13.0
	18	100.2	6.6	103.1	4.4	107.1	9.6	96.0	9.2
	180	104.8	5.5	101.5	6.6	92.8	3.5	101.4	7.8
Sempervirine	0.4	105.3	5.4	98.3	6.9	107.8	7.5	108.5	10.5
	18	99.9	5.3	99.7	2.9	100.5	10.8	95.4	8.3
	180	101.2	1.2	101.5	5.2	94.1	9.5	88.4	8.1

**Table 5:** Toxicokinetic parameters of 11 Gelsemium alkaloids after intravenous administration in rats (0.1 mg/kg for each alkaloid).

Compound	AUC_(0-__*t*__)_	AUC_(0-∞)_	MRT_(0-__*t*__)_	MRT_(0-∞)_	*t*_1/2*z*_	Vz	CL_*z*/*F*_	*C*_max_
	ng/mL∗h	ng/mL∗h	h	h	h	L/kg	L/h/kg	ng/mL
Humantenirine	73.1 ± 19.5	73.2 ± 19.5	1.4 ± 0.1	1.4 ± 0.1	0.9 ± 0.3	2.0 ± 0.8	1.4 ± 0.4	43.7 ± 6.9
Humantenine	14.7 ± 3.7	14.0 ± 3.7	1.2 ± 0.1	1.2 ± 0.1	1.5 ± 0.3	15.9 ± 4.9	7.6 ± 2.3	11.5 ± 3.6
Akuammidine	4.7 ± 1.31.3	4.7 ± 1.2	1.0 ± 0.4	1.2 ± 0.5	2.3 ± 1.2	84.0 ± 63.3	22.5 ± 6.5	7.8 ± 2.1
Gelsevirine	73.8 ± 12.9	122.7 ± 107.2	7.2 ± 1.6	22.0 ± 31.1	15.8 ± 8.1	14.2 ± 6.3	1.2 ± 0.5	13.2 ± 3.9
Rankinidine	2.2 ± 0.8	2.2 ± 0.8	1.0 ± 0.2	1.1 ± 0.2	0.9 ± 0.1	67.5 ± 22.7	52.7 ± 22.4	2.3 ± 1.2
n-Methoxyanhydrovobasinediol	3.1 ± 0.7	3.6 ± 0.7	1.8 ± 0.5	3.3 ± 2.0	2.7 ± 1.9	106.9 ± 72.2	28.3 ± 5.7	2.4 ± 1.4
Gelsenicine	7.5 ± 4.9	7.6 ± 4.9	0.8 ± 0.2	0.8 ± 0.2	1.4 ± 1.0	3.2 ± 2.2	1.7 ± 0.8	9.4 ± 4.9
Gelsemine	1.7 ± 0.5	2.0 ± 0.6	2.1 ± 0.4	3.7 ± 2.1	3.0 ± 1.7	216.2 ± 104.3	53.0 ± 16.3	1.1 ± 0.5
Koumine	7.9 ± 2.3	8.5 ± 2.7	0.8 ± 0.2	1.2 ± 0.5	1.1 ± 0.6	21.5 ± 12.6	13.6 ± 7.3	10.3 ± 3.6
Koumidine	6.7 ± 2.9	6.9 ± 2.9	1.8 ± 0.3	2.1 ± 0.5	2.2 ± 1.0	54.4 ± 34.7	17.1 ± 7.5	5.4 ± 2.1
Sempervirine	25.7 ± 6.8	29.4 ± 9.7	6.5 ± 2.1	9.7 ± 4.9	7.7 ± 3.8	37.2 ± 12.1	3.7 ± 1.2	9.3 ± 4.4

## Data Availability

The data used to support the findings of this study are included within the article.
